# Quantitative membrane proteomics reveals a role for tetraspanin enriched microdomains during entry of human cytomegalovirus

**DOI:** 10.1371/journal.pone.0187899

**Published:** 2017-11-09

**Authors:** Kasinath Viswanathan, Marieke C. Verweij, Nessy John, Daniel Malouli, Klaus Früh

**Affiliations:** Vaccine and Gene Therapy Institute, Oregon Health and Science University, Beaverton, Oregon, United States of America; University of St Andrews, UNITED KINGDOM

## Abstract

Human cytomegalovirus (HCMV) depends on and modulates multiple host cell membrane proteins during each stage of the viral life cycle. To gain a global view of the impact of HCMV-infection on membrane proteins, we analyzed HCMV-induced changes in the abundance of membrane proteins in fibroblasts using stable isotope labeling with amino acids (SILAC), membrane fractionation and protein identification by two-dimensional liquid chromatography and tandem mass spectrometry. This systematic approach revealed that CD81, CD44, CD98, caveolin-1 and catenin delta-1 were down-regulated during infection whereas GRP-78 was up-regulated. Since CD81 downregulation was also observed during infection with UV-inactivated virus we hypothesized that this tetraspanin is part of the viral entry process. Interestingly, additional members of the tetraspanin family, CD9 and CD151, were also downregulated during HCMV-entry. Since tetraspanin-enriched microdomains (TEM) cluster host cell membrane proteins including known CMV receptors such as integrins, we studied whether TEMs are required for viral entry. When TEMs were disrupted with the cholesterol chelator methyl-β-cylcodextrin, viral entry was inhibited and this inhibition correlated with reduced surface levels of CD81, CD9 and CD151, whereas integrin levels remained unchanged. Furthermore, simultaneous siRNA-mediated knockdown of multiple tetraspanins inhibited viral entry whereas individual knockdown had little effect suggesting essential, but redundant roles for individual tetraspanins during entry. Taken together, our data suggest that TEM act as platforms for receptors utilized by HCMV for entry into cells.

## Introduction

The β-herpesvirus HCMV establishes asymptomatic persistent infection in immune competent adults. While most of the world’s population is infected with this virus, with more than 80% prevalence in developing countries [1], HCMV infection is of particular clinical importance in immunocompromised individuals. The virus can cause deafness and mental retardation in neonates [2, 3], retinitis and blindness in AIDS patients [4], graft versus host disease following bone marrow transplantations and disseminated disease and graft rejection in solid organ transplantations [5]. HCMV is the largest of the characterized human herpesviruses containing a ~236kb genome that encodes approximately 170 open reading frames [6], of which only 45 are essential for virus replication *in vitro* [7]. The viral proteins are expressed in three sequential cascades, immediate early (IE), early (E) and late (L), whereby the late genes can be further subdivided in early-late (E/L) and true late (L) genes. Many of these proteins interact with and modulate protein networks of the host cell [8]. Functional genomics approaches such as computational network analysis, global transcriptomics, proteomics of host cell-associated and secreted proteins as well as metabolomics are increasingly being used to obtain a comprehensive picture of interactions between virus and host proteins, and to determine the importance of individual interactions in controlling viral entry, replication and egress [9]. Initially, DNA microarrays were used to predict changes in the host cell proteome and these analyses revealed differential expression of hundreds of host transcripts during HCMV infection [10]. However, protein levels do not necessarily reflect transcription levels and recent efforts are targeted towards generating more direct evidence in virus-induced changes in the proteome and metabolome of the host cells. To monitor abundance and post-translational modification, one approach is to use multiple antibodies against cellular proteins. For instance, by high-throughput Western blot screening of antibodies against 1009 cellular proteins, the focal-adhesion associated proteins Hic-5, paxillin and α-actinin were identified as targets of host protein manipulation by HCMV [11]. In addition, proteomics were used to discover binding partners for single viral proteins [12–14] and co-immunoprecipitations revealed interactions between the HCMV virion proteins pp28, pp150 and gB and cellular proteins indicating possible involvement in HCMV particle assembly and egress [12]. For example, pp28 was shown to associate with members of the ESCRT pathway [12], which was previously demonstrated to be essential for efficient HCMV virion assembly [15]. Proteomics was also successfully applied to determine the complete proteome of purified virions [16–18]. At a more global level, mass spectrometry was used to identify HCMV-induced changes in host metabolites using metabolomics [19, 20]. Furthermore, virus-induced changes in the secretome of infected cells were determined by proteomics [21]. Large scale protein identification was also employed to demonstrate changes in the protein levels of host proteins in endoplasmic reticulum-mitochondrial contacts, known as mitochondria-associated membranes [22]. The virus-induced proteins included multiple host proteins required for viral assembly, showing direct manipulation of the host cell by the virus on the protein level [22]. Recently, a multiplexed tandem mass tag-based mass spectrometry technique called quantitative temporal viromics was employed to study the interactions between HCMV and host proteins at the plasma membrane as well as in the entire cell in more detail [23]. This study did not only illuminate the effect of HCMV infection on the expression levels of over 8,000 different cellular proteins over time, it also shed light on the kinetics of the expression of canonical as well as some non-canonical viral genes. Data generated by this study support the existence of at least one new kinetic class of viral gene expression in addition to the established IE, E, E/L-L expression cascades. Furthermore, using this technique, new ligands for NK- and T-cells were predicted, potentially opening the door for novel therapeutic targets [23].

We previously used stable isotope labeling with amino acids in cell culture (SILAC) together with MUlti Dimensional protein Identification Technology (MuDPIT) to successfully identify differentially expressed proteins in membranes of cells expressing viral or cellular membrane ubiquitin ligases [24, 25]. Others have used this technique for high complexity samples [26] and to identify direct protein targets of small molecules [27]. In addition to identifying host cell proteins affected by specific viral proteins, this method is also suited to identify changes in protein abundance upon viral infection of tissue culture cells. Therefore, we applied this approach to study the changes in host protein expression in human fibroblasts infected with HCMV. We identified specific host cell membrane proteins that changed in their abundance upon HCMV infection and confirmed their differential expression using independent experimental approaches. Subsequent analysis revealed a role of tetraspanins in HCMV-entry.

## Materials and methods

### Antibodies and reagents

Monoclonal antibodies to GAPDH (6C5), CD44 (HCAM F4 and DF1485), CD151 (11G5a), caveolin-1 (7C8), and ITGB1 (4B7R) were purchased from Santa Cruz Biotechnologies; monoclonal antibodies to GRP78 (40/BiP), CD98 (UM7F8), and transferrin receptor (TfR, M-A712) were purchased from BD Biosciences; the rat monoclonal BAP31 was purchased from Affinity BioReagents (CC-1); the CD59 monoclonal antibody was purchased from GeneTex (8D2B8); CD81 antibodies were purchased from BD Biosciences (JS-81, monoclonal, used for flow cytometry and immunofluorescence assays) and Santa Cruz Biotechnologies (H-121, polyclonal, used for Western blot under reducing conditions); monoclonal antibodies against EGFR were obtained from BD Biosciences (EGFR.1) and Millipore (Ab-5); monoclonal CD9 antibodies were obtained from BD Biosciences (RPM.7) and Serotec (MM2/57). Tetraspanin-specific monoclonal antibodies to CD151 and CD9 were a kind gift from Dr. Fedor Berditchevski (School of Cancer Sciences, University of Birmingham, UK) [28]. Rabbit polyclonal antisera to HCMV pp28 and IE1 were a kind gift from Dr. Jay Nelson (Vaccine and Gene Therapy Institute, Oregon Health and Science University, Beaverton, Oregon, USA). The monoclonal antibody W6/32 recognizing the complex of heavy chain and β2M was used to detect MHC class I (MHC I). Foscarnet (sodium phosphonoformate tribasic) was purchased from Sigma-Aldrich Chemicals. The cytokine IFNβ was obtained from PBL Assay Science and the dsRNA mimic poly (I:C) was purchased from Amersham biosciences.

### Virus and cell culture

Primary human foreskin fibroblast (HFF) cells were obtained from ATCC and cultured in DMEM supplemented with 10% fetal bovine serum (FBS), L-Glutamine and 100 units of penicillin/streptomycin in a humidified incubator with 5% CO_2_ at 37°C. HFFs used in this study were passaged between 8–25 times. The HCMV strains AD169, Toledo and Powers were a gift from Dr. Jay Nelson (Vaccine and Gene Therapy Institute, Oregon Health and Science University, Beaverton, Oregon, USA). HFFs were infected with AD169, Toledo or Powers at an MOI of 3 unless otherwise mentioned. The HCMV strain TB40-GFP was kindly provided by Dr. Felicia Goodrum (Department of Immunobiology, University of Arizona, Tucson, Arizona, USA) and used to infect HFFs at an MOI of 5. To generate viral stocks, HFFs were infected at an MOI of 0.5 and harvested when they displayed maximal cytopathic effect. The cells were separated from the supernatant by two consecutive rounds of centrifugation at 7.500 x *g* for 15 min. The clarified medium was layered over a sorbitol cushion (20% D-sorbitol, 50 mM Tris [pH 7.4], 1 mM MgCl_2_), and the virus was pelleted by centrifugation at 64.000 x *g* for 1h at 4°C in a Beckman SW28 rotor. The virus pellet was resuspended in DMEM with 10% FBS and penicillin/streptomycin. UV-inactivated AD169 was prepared by placing 200 μl of virus into the wells of a 12 well plate followed by two cycles of irradiation for 30 sec with 1000 μeV. HSV-1 strain F was provided by Dr. David Johnson (Oregon Health and Science University, Portland, Oregon, USA) and HFFs were infected with the virus at an MOI of 10. Vesicular stomatitis virus (VSV Indiana) was purchased from ATCC (VR-1238) and was used at an MOI 10. The vaccinia virus strain Western Reserve expressing GFP was provided by Dr. Gary Thomas (University of Pittsburgh, School of Medicine, Pittsburgh, USA). The recombinant cowpoxvirus CPXVΔ203 expressing GFP was a kind gift from Dr. Wayne Yokoyama and was previously described [29]. Both viruses were used at on MOI of 5.

### Isotope labeling and plasma membrane protein isolation

For quantitative analysis of changes in the plasma membrane proteome upon HCMV infection, SILAC was used as described [25]. Briefly, the HFFs were labeled with stable amino acid isotopes using labeling medium (DMEM) lacking the amino acids L-lysine and L-leucine (Invitrogen). Medium was supplemented with 10% dialyzed FBS (Hyclone), penicillin/streptomycin, and either isotopically light L-lysine and L-leucine (Sigma-Aldrich Chemicals) or isotopically heavy L-lysine (U-^13^C_6_, 98%; U-^15^N_2_, 98%) and L-leucine (U-13C6, 98%; 15N, 98%) (Cambridge Isotope). HFFs were maintained in labeling medium for 6 population doublings prior to initiation of the infection. HFFs were infected with AD169 for 24h before isolating plasma membrane proteins and the experiment was repeated with heavy and light isotopes reversed. The cells were harvested by scraping, washed twice in PBS, resuspended in PBS containing 5 mM EDTA, and lysed by douncing. Unlysed cells and debris were cleared from the lysate by centrifugation for 5 min at 3.000 x *g*. The cleared lysates were separated into membrane and soluble fractions by centrifugation for 30 min at 45.000 x *g*. The membrane fraction was resuspended in PBS, sonicated and separated over a discontinuous sucrose gradient (2 M, 1.6 M, 1.25 M, 1.2 M, and 0.8 M) by centrifugation for 2.5h at 25.000 rpm (Sorvall SW-41 rotor). The band corresponding to the plasma membrane (0.8–1.2 M inter phase) fraction was removed, diluted in 5 mM Tris-EDTA buffer (pH 8.0) and centrifuged for 30 min at 45.000 x *g* to pellet the proteins contained in each fraction. Pellets were washed for 30 min in 50 mM sodium bicarbonate (pH 11.5) and centrifuged for 30 min at 45.000 x *g*, followed by a second wash in 50 mM ammonium bicarbonate (pH 8.5) and further centrifugation for 30 min at 45.000 x *g*. Final pellets were resuspended in 8.0 M deionized urea and 50 mM ammonium bicarbonate (pH 8.5) and protein levels quantitated using the Bio-Rad Protein Assay (Bio-Rad). Samples were reduced with dithiotreitol (DTT, Sigma-Aldrich Chemicals) and alkylated with iodoacetamide (Sigma-Aldrich Chemicals) prior to overnight digestion with trypsin (Promega).

### Chromatography, mass spectrometry, and bioinformatics

Peptide mixtures were analyzed using ESI-MS/MS coupled with 2D-LC. 22 μg of the sample was loaded onto an Opti-Pak capillary SCX trap cartridge (Optimize Technologies) and eluted stepwise (using 12.5, 25, 37.5, 50, 62.5, 75, 87.5, 100, 112.5, 125, 200, 300, or 450 mM ammonium acetate in 0.1% formic acid) onto a reverse phase C-18 capillary column (180 μm X 100 mm, BioBasic-18; Thermo Electron). Peptides were then eluted using an acetonitrile gradient (5%, 5 min; 5%–40%, 75 min; 40%–90%, 10 min) into a ProteomeX LCQ Deca XP Plus mass spectrometer (Thermo Electron) equipped with a low-flow (1 μl/min) electrospray source. The instrument was set to trigger data dependent MS/MS acquisition of the three most intense ions detected during the MS survey scan when total ion current per MS survey scan exceeded 5.0 X 10^5^ counts. Proteins were identified by analyzing tandem mass spectra with the Sequest algorithm (Thermo Electron) as described by Yates et al. [30] using the human subset of the UniProt/Swiss-Prot protein database spiked with HCMV proteins. The search results were further analyzed using Peptide Prophet [31]. SILAC ratios were determined using the ASAPRatio algorithm (ISB, Seattle) [32]. Multiple peptides derived from a single protein were included if Peptide Prophet probability was greater than or equal to 0.85. All positive results were manually verified.

### Immunofluorescence assays

Cells were grown in 35 mm dishes and infected with HCMV AD169 at an MOI of 0.5. At 24 hpi the cells were washed with PBS, fixed with 2% paraformaldehyde for 10 min at room temperature (RT), and permeabilized with 0.2% Triton X-100 for 5 min at RT. Non-specific binding sites were blocked with 3% horse serum in PBS for 15 min at 37°C. The fixed cells were incubated with primary antibody diluted in blocking solution for 2h at 4–8°C. Then the cells were washed and incubated with the appropriate secondary antibody diluted in blocking solution for 1h at 4–8°C. After washing three times with PBS, cells were fixed again in 2% paraformaldehyde and washed twice with PBS. Coverslips were mounted onto slides and covered with Vectashield H-1200 with DAPI (Vector Laboratories).

### Flow cytometry

HFFs were removed from tissue culture dishes with 0.05% trypsin-EDTA (Invitrogen), washed with ice-cold PBS, and fixed with 2% paraformaldehyde. For surface staining HFFs were directly incubated with the indicated primary antibody in PBS or PBA (PBS supplemented with 0.05% sodium azide and 1% bovine serum albumin) for 30–60 min at 4–8°C. As isotype control, cells were incubated with a similar concentration of normal mouse IgG (Invitrogen). The cells were washed twice with ice-cold PBS or PBA and incubated with PE-conjugated anti-mouse secondary antibody (Dako) or with Alexa Fluor 647 chicken anti-mouse (Life Technologies) in PBS or PBA for 30–60 min at 4–8°C. The cells were washed twice before resuspending in 1% paraformaldehyde and analysis with a BD Biosciences FACSCalibur or LSR II flow cytometer. For intracellular staining, cells were permeabilized using 0.5% saponin. Primary and secondary antibodies were resuspended in 0.5% and washes were performed with 0.5% saponin as well.

### Cell surface protein biotinylation

Cell surface proteins were biotinylated with EZ-Link NHS-SS-biotin following the manufacturer’s protocol (Pierce). Briefly, cells were washed three times with ice-cold PBS, and primary amines of the membrane proteins exposed to the exterior of the cells were biotinylated with NHS-SS-biotin for 30 min at 4°C. The cells were washed and lysed immediately with a nonionic detergent. Labeled proteins were isolated with immobilized NeutrAvidin agarose beads (Pierce). The bound proteins were released by incubating the resin with sodium dodecyl sulfate-polyacrylamide gel electrophoresis (SDS-PAGE) sample buffer containing 50 mM DTT (Sigma-Aldrich Chemicals).

### Adenovirus expression of viral proteins

The recombinant adenoviruses expressing HCMV pp65, pp71 or gB, provided by Dr. Wade Bresnahan, (University of Minnesota, Minneapolis, Minnesota, USA), and IE1 or IE2, provided by Dr. Dan Streblow (Vaccine and Gene Therapy Institute, Oregon Health and Science University, Beaverton, Oregon, USA) were produced as previously described [33]. The vectors used contain a tetracycline-responsive promoter and require the addition of a tetracycline transactivator (tTA) [34], which was provided by co-infecting with an adenovirus encoding the tetracyclin-transactivator (AdTA). HFFs were infected with the purified adenoviruses expressing the HCMV proteins at an MOI of 100. AdTA was used at an MOI of 10. After 16-24h post infection the cells were harvested and analyzed for CD81 expression by flow cytometry.

### MβCD treatment

HFFs were treated with the indicated quantities (0.4, 2.0, 8.0 and 25 mM) of Methyl β-D-Cyclodextrin (MβCD, Sigma-Aldrich Chemicals) at 37°C for 1h while rocking before infecting with HCMV. The cells were harvested at the times indicated in each experiment and analyzed. For experiments separating detergent soluble and insoluble fractions, cells were harvested and treated with ice-cold 1% Triton X-100. Cells were centrifuged twice to clear nuclei and unlysed cells, which was followed by a final spin at 45.000 rpm, 4°C for 1h. The supernatant and precipitates were separated and probed for the test proteins. For flow cytometry analysis HFFs were treated with MβCD for 1h at 37°C and then washed with PBS and fixed with paraformaldehyde (2%) followed by staining as described above. For cholesterol replenishing, the cells were treated with MβCD for 1h at 37°C followed by repeated washing and addition of 20 μg cholesterol per well. After incubating the cells for 2h, surface levels of tetraspanins were determined.

### RNA isolation and semiquantative PCR

Total mRNA was isolated from cells and purified using the RNeasy RNA isolation kit (Qiagen). Single-stranded cDNA was made from total RNA using random hexamers (TaKaRa) to prime first-strand synthesis by SuperScript III Reverse Transcriptase (Life Technologies) as recommended by the manufacturer. Transcript levels were determined by quantitative real-time reverse transcription PCR (qPCR), using SYBR green dye incorporation and AmpliTaq Gold DNA polymerase (Applied Biosystems) in an ABI Prism 7900HT sequence detection system (Applied Biosystems). The following primers were used: CD44 Fw: 5’-CGGACACCATGGACAAGTTT-3’; CD44 Rev: 5’-GAAAGCCTTGCAGAGGTCAG-3’; CD81 Fw: 5’-CATCTACATCCTCATCGCTG-3’; CD81 Rev: 5’-CATCCTTGGCGATCTGGTC-3’; GRP78 Fw: 5’-GGATCATCAACGAGCCTAC-3’; GRP78 Rev: 5’-GTCCTTCCTGACATCTTTGC-3’; β-actin Fw: 5’-TCCCTGGAGAAGAGCTACGA-3’; β-actin Rev: 5’-AGCACTGTGTTGGCGTACAG-3’; GAPDH Fw: 5’-AATCCCATCACCATCTTCCA-3’; and GAPDH Rev: 5’-TGGACTCCACGACGTACTCA-3’. The comparative threshold cycle method was used to derive the change in gene expression between different treatments, using β-actin or GAPDH as an internal standard.

### siRNA treatment

HFFs were reverse transfected using 3 μl of Lipofectamine-2000 (Invitrogen) per 1x10^5^ cells and 20–40 nM of siRNA for 64h and then infected with HCMV. The total concentration of siRNA was kept the same between the different samples. siRNAs against GAPDH were obtained from Dharmacon RNAi technologies (SMARTpool ON-TARGETplus L-004253-00-0005). siRNAs specifc for CD81 (sc-35030), CD9 (sc-35032) and CD151 (sc-42829 were obtained from Santa Cruz biotechnologies.

### Virus attachment, entry, and early gene expression assays

To monitor virus attachment, HFFs were cooled down to 4°C before incubation with AD169 (MOI of 0.5) for 1h at 4°C while rocking. Cells were washed three times with ice-cold PBS and lysed in loading buffer for Western blot analysis. To monitor viral entry, HFFs were treated with AD169 (MOI of 0.5) at 37°C for 1h while rocking. The cells were washed with PBS followed by two washes with acidic citrate buffer (pH 2.5) and two washes with PBS. The cells were harvested and tested for the presence of pp65 using Western blot analysis. To measure early gene expression, cells were treated with AD169 (MOI of 3.0) for 8h and analyzed for IE1 protein expression. GAPDH was used as a control throughout to calculate the relative ratio of pp65 or IE1 in the samples.

### Western blotting

HFFs were lysed in Laemmli Buffer (10% glycerol, 5% β-mercaptoethanol, 2% SDS, 50 mM Tris [pH 6.8], and 0.02% bromophenol blue), and separated by 10% SDS-PAGE. The proteins were transferred to polyvinylidene difluoride membranes (Waters Ltd.) and probed with the indicated primary antibodies for 1h at RT or overnight at 4°C. This was followed by incubation with horseradish peroxidase-conjugated secondary antibodies specific for mouse IgG (Santa Cruz Biotechnologies). Staining of the membranes was visualized by using the SuperSignal West Pico Chemiluminescence substrate (Thermo Scientific). The films were scanned and the intensity of the bands was measured using the Image J 1.42q software (http://imagej.nih.gov/ij/).

## Results

### Quantitative membrane proteomics analysis reveals differentially expressed host cell membrane proteins in HCMV-infected human fibroblasts

**Fig 1:**
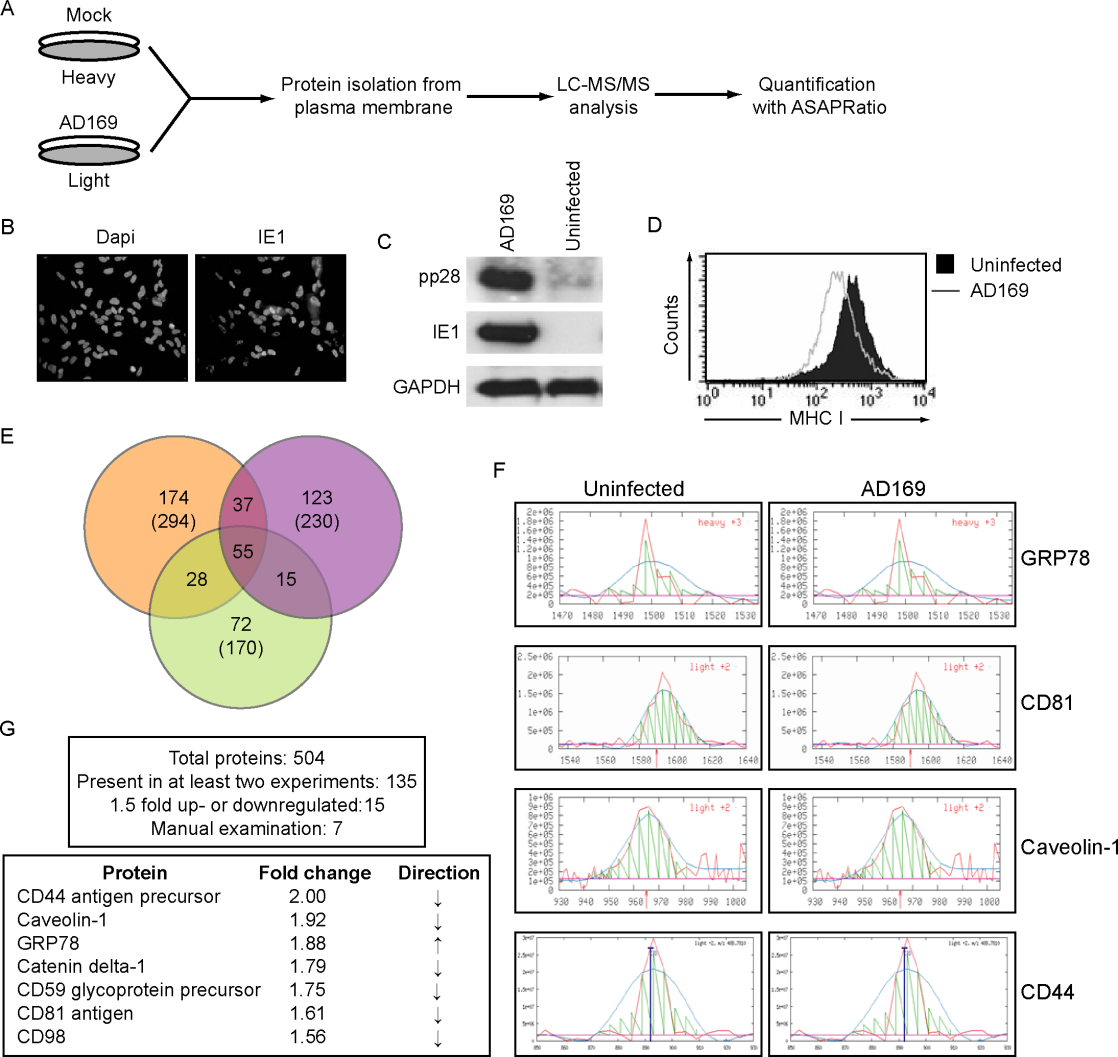
Multiple host cell surface proteins show altered expression levels after HCMV infection. (A) Schematic overview of the proteomic analysis. HFFs were infected with AD169 at an MOI of 3. 24h later, IFA was used to verify the infection levels by intracellular IE1 staining. (B), by SDS-PAGE and Western blot analysis of whole cell lysates for pp28 and IE1 (C) and by monitoring MHC class I downregulation from the cell surface of infected cells using flow cytometry (D). (E) The results of three independent SILAC experiments are summarized. (F) Shown is the area measurement of the eluted peaks corresponding to the peptides of four of the selected proteins. (G) The selected seven proteins for follow-up studies are listed with their respective fold changes.

HCMV-induced changes in protein abundance at the plasma membrane were analyzed in infected HFFs using quantitative membrane proteomics. HFFs were metabolically labeled with different stable isotopes of carbon and nitrogen incorporated into the amino acids L-Leucine (L-Leu) and L-Lysine. L-Leu was selected instead of the more commonly used Argenine (Arg), because L-Leu is more abundant than Arg and will likely increase peptide per protein detection. In addition, Arg might be converted to proline resulting in a different peptide mass. The cells grown in heavy media were uninfected and the cells grown in light media were infected with the laboratory-adapted HCMV strain AD169 for 24h (Fig 1A). HCMV-infection of over 90% was confirmed by staining the cells for expression of IE1 by immune fluorescence microscopy (IFA) (Fig 1B). Viral gene expression was also verified by Western blotting for IE1 and the L protein pp28 (Fig 1C). To further control for HCMV-infection we analyzed the cell surface levels of MHC class I (MHC I) using flow cytometry. HCMV downregulates MHC I by expression of the US6-family of viral inhibitors of antigen presentation [35, 36]. The reduction observed on HCMV-infected cells confirmed productive infection (Fig 1D). At 24h post infection (hpi), the cells were harvested and membrane proteins were isolated by cell fractionation. The proteins were trypsinized and analyzed using tandem 2D-LC-MS/MS (Fig 1A). In three independent experiments, we identified a total of 504 proteins that changed in their abundance when using the algorithm ASAPRatio (Fig 1E). The data of the individual experiments can be found in S1–S3 Tables (S1–S3 Tables). All results are combined in S4 Table (S4 Table). From the initial dataset we selected seven proteins for follow-up studies based on the following criteria: a minimum 1.5 fold change in expression, differential expression in at least two of the three replicates as well as manual curation to confirm the identity of the peptide through fragmentation pattern in the mass spectrum and the area measurement of the eluted peaks corresponding to the peptides (Fig 1F). Among the seven selected proteins, only glucose-regulated protein (GRP) 78 showed an HCMV-induced increase in protein expression (1.88 fold), whereas CD44 (2.00 fold), caveolin-1 (1.92 fold), catenin delta-1 (δ-catenin) (1.79 fold), CD59 (1.75 fold), CD81 (1.61 fold) and CD98 (1.56 fold), were downmodulated by HCMV infection (Fig 1G).

### Validation of membrane protein downregulation by HCMV

GRP78 is an endoplasmic reticulum (ER) resident chaperone that has been previously shown to be upregulated during HCMV infection [37]. It was observed that the HCMV US2 and US11 proteins require GRP78 for degradation of MHC I [38]. GRP78 is also essential for the structure and function of the assembly compartments during HCMV infection [39]. Although GRP78 is predominantly present in the ER, it is also detectable at the cell surface where GRP78 is known to act as a receptor for α2-macroglobulin [40]. Our proteomics results thus confirm the upregulation of GRP78 in cell membranes during HCMV infection.

**Fig 2:**
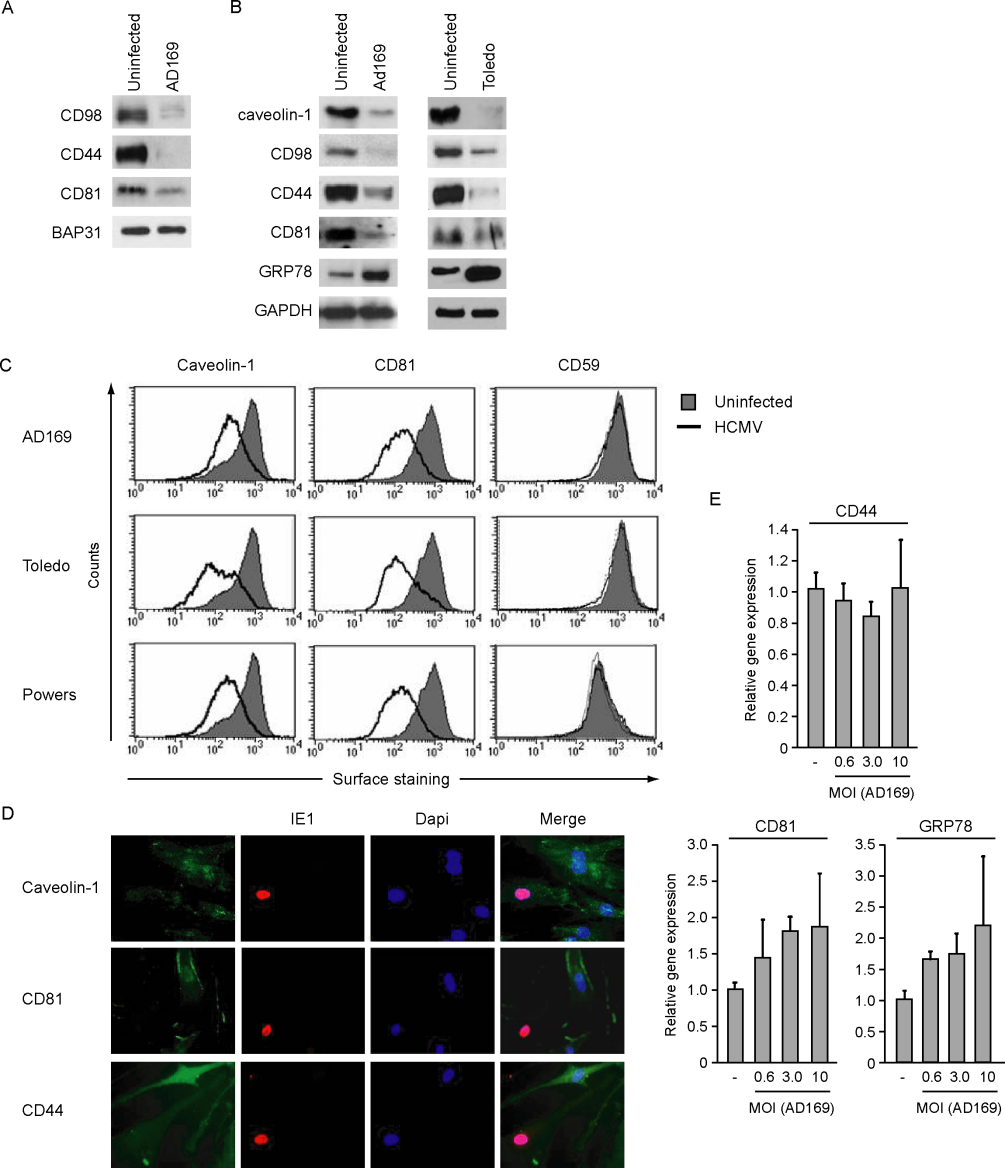
Validation of membrane protein downregulation by HCMV. HFFs were left uninfected or infected with the indicated HCMV strains at an MOI of 3 for 24h. (A) Cell surface proteins were biotinylated and isolated using NeutrAvidin agarose beads. Bound proteins were analyzed by SDS-PAGE and Western blot using the indicated antibodies. (B) Total changes in expression levels of the specified proteins were analyzed in whole cell lysates by SDS-PAGE and Western blot using specific antibodies. Cell surface downregulation of a selection of the proteins was also studied using flow cytometry (C) and IFA (D). Experiments were repeated two times and the result of one representative experiment is shown. For caveolin-1 staining cells were permeabilized with 0.5% Saponin for flow cytometry and 0.2% Triton X-100 for IFA. HCMV IE1 co-staining was performed to identify HCMV infected cells. (E) HFF cells were infected with increasing MOI of HCMV AD169 and at 24 hpi mRNA levels of CD44, CD81 and GRP78 were assessed using by qPCR. The results displayed are an average of three independent experiments that each included three replicates.

The downregulated proteins CD81, CD44 and CD98 are transmembrane proteins with extracellular domains. To independently confirm the changes in protein abundance identified by proteomics, we analyzed the surface expression of these proteins by cell surface biotinylation, flow cytometry and IFA in uninfected and HCMV-infected HFFs. The individual assays were chosen depending on the commercial availability of antibodies working in a given experimental setting. Cell surface biotinylation confirmed an apparent reduction in surface levels for CD98, CD44, and CD81 in cells infected with AD169 (Fig 2A). In contrast, the control protein BAP31 did not show a consistent change in our proteomics experiment, which was confirmed in the biotinylation experiment (Fig 2A). BAP31,one of the most abundant ER-resident proteins that facilitates the intracellular transport of transmembrane proteins, including tetraspanins [41], but the protein can also be found on the cell surface [42]. Since δ-catenin and caveolin-1 are cytoplasmic membrane-associated proteins and are hence not expressed on the cell surface, surface biotinylation results were not included for these proteins. Nevertheless, HCMV infection with AD169 did reduce the steady state levels of caveolin-1 as well as CD98, CD44, and CD81 as shown by Western blotting of whole cell lysates. Similar results were observed with the low-passage Toledo isolate (Fig 2B). An HCMV-induced upregulation in GRP78 was also observed in the whole cell lysates **(**Fig 2B**)**. Using flow cytometry we studied intracellular levels of caveolin-1 and cell surface expression of CD81 and CD59 (Fig 2C). These data demonstrate that the HCMV strains AD169, Toledo and Powers all downregulate caveolin-1 and CD81 to a similar extent, suggesting that the modulation of these host proteins is strain-independent. Although CD59 was initially identified in our mass spectrometry as a potential candidate for HCMV induced downmodulation (Fig 1G), this was not confirmed by flow cytometry analysis (Fig 2C). The IFA results showed diminished staining of caveolin-1, CD81 and CD44 in AD169-infected cells, which are identified by IE1 staining (Fig 2D). In contrast, neighboring non-infected cells displayed clear expression of the indicated proteins (Fig 2D). Previously reported DNA microarray analysis results of HCMV-infected HFFs suggested that the transcription of caveolin-1, CD81, CD44, and CD98 are not affected by the virus [43]. Examining mRNA levels of CD44, CD81 and GRP78 using semiquantative PCR in HFFs infected with an increasing MOI of HCMV AD169 did not show a significant change (Fig 2E), indicating that the downmodulation from the cell surface as well as the overall decrease in whole cell lysate protein levels of the examined proteins are due to post-transcriptional events.

### CD81 is downmodulated by HCMV

**Fig 3:**
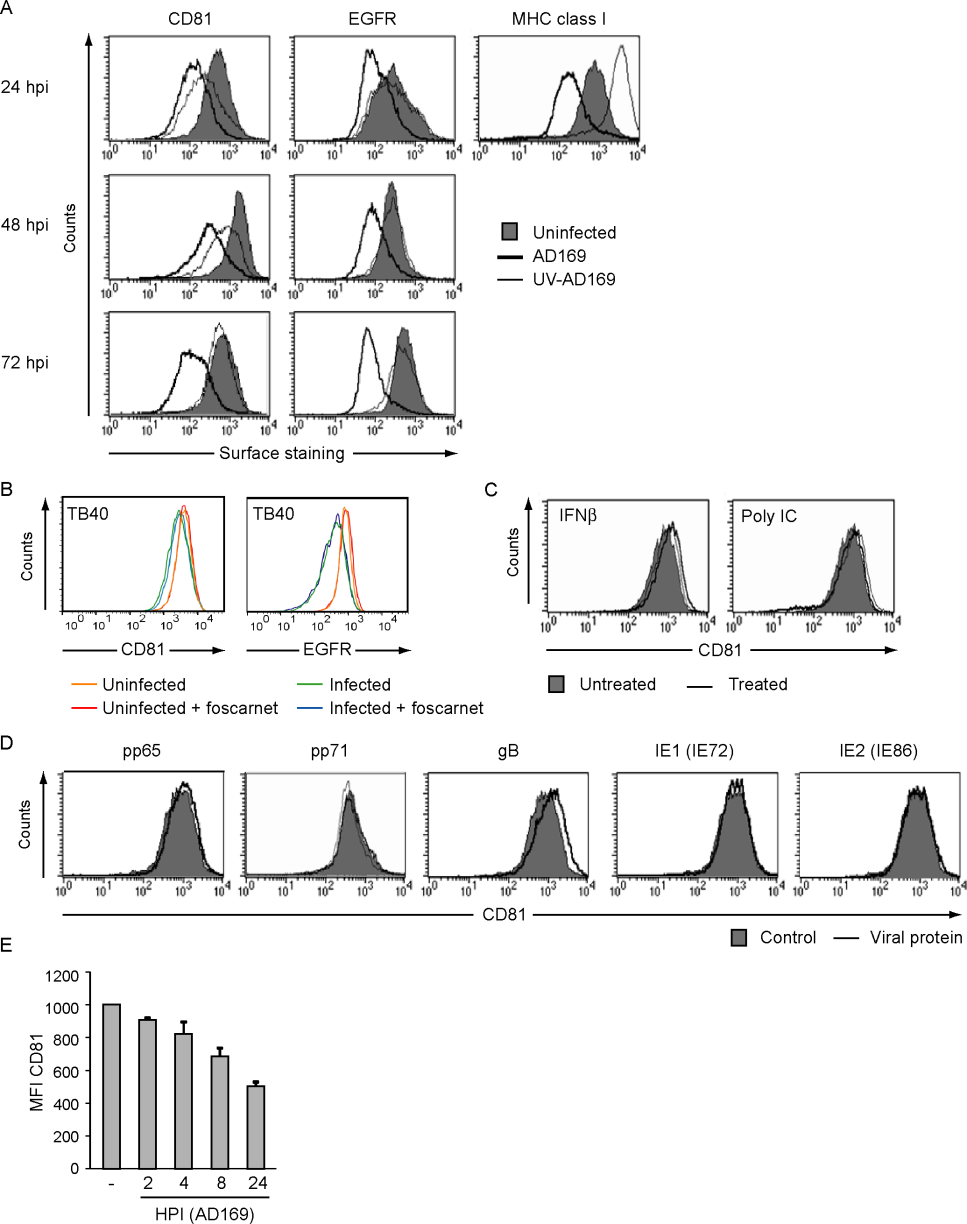
CD81 downregulation occurs in the absence of viral gene expression. (A) HFFs were left uninfected, or infected with UV-inactivated AD169 or AD169 at an MOI of 3. At 24, 48 and 72 hpi surface expression of CD81 and EGFR was determined by flow cytometry. Upregulation of MHC I by UV-inactivated HCMV and downregulation of MHC I by HCMV were confirmed by flow cytometry at 24 hpi. (B) HFFs were infected with HCMV TB40-GFP in the presence or absence of foscarnet. At 24 hpi the cell surface expression of CD81 and EGFR was assessed using flow cytometry. (C) HFFs were treated with IFNß or Poly I:C for 8h and CD81 cell surface expression was examined by flow cytrometry. (D) HFFs were infected with adenoviruses expressing the specified HCMV proteins or with a control adenovirus expressing the tetracyclin transactivator for 24h, after which CD81 cell surface expression was monitored by flow cytometry. All experiments were repeated two times and the result of one representative experiment is shown. (E) HFFs were infected with AD169 at an MOI of 3 and the cell surface expression of CD81 was examined at 2, 4, 8 and 24 hpi by flow cytometry. Results shown are an average of two independent experiments.

To determine whether HCMV-mediated reduction of CD81 was due to expression of viral gene products, we monitored its cell surface expression in HFFs infected with UV-inactivated AD169 (UV-AD169). We also stained for epidermal growth factor receptor (EGFR), which has been implicated in HCMV entry [44]. Downregulation of MHC I by HCMV requires viral gene expression [45]. Therefore, UV-inactivation of HCMV was verified by monitoring MHC I expression on uninfected, AD169- and UV-AD169-infected cells by flow cytometry. The increased MHC I expression observed in UV-AD169-infected cells confirms inactivation of virus (Fig 3A, right panel). EGFR levels were not affected by UV-AD169, indicating a requirement for viral gene expression and not a phenotype directly connected to viral entry. In contrast, CD81 was still downregulated by UV-AD169 and displayed a transient phenotype reverting back to uninfected levels at around 72 hpi (Fig 3A). Thus, it appears that CD81 downregulation is bi-phasic with the first phase being independent of gene expression whereas the second phase is independent of viral late gene expression. To examine whether the downregulation of CD81 and EGFR is due to viral early or late genes we treated infected cells with foscarnet, a drug that inhibits viral DNA replication and thus late gene expression [46]. Treatment with foscarnet did not affect HCMV-mediated downregulation of CD81 and EGFR (Fig 3B), indicating that EGFR downregulation is mediated by an IE or E gene. We confirmed Foscarnet activity by demonstrating the inhibition of expression of glycoprotein D in herpes simplex virus (HSV)-1-infected cells.

Infection of HFFs with UV-HCMV induces a strong innate immune response, predominated by interferon-stimulated genes (ISGs) [47]. This is illustrated by surface levels of MHC I that are upregulated in cells infected with UV-AD169 due to transcriptional induction via the IFN pathway (Fig 3A, right panel). To determine whether the reduction in CD81 surface levels is induced by ISG expression HFFs were treated with IFN-β or transfected with the dsRNA mimic Poly I:C for 8h to activate innate immune signaling. No change in the expression of CD81 was observed by flow cytometry in response to either treatment (Fig 3C) suggesting that early stage CD81-downregulation is not induced by ISG expression.

During viral entry, proteins of the viral tegument are released into the cytoplasm, including two major tegument proteins pp65 and pp71 that perform immediate intrinsic and innate immune evasion functions [48–50]. To study whether these proteins play a role in the observed CD81 downregulation, HFFs were co-infected with adenoviruses expressing the indicated viral proteins under a tetracycline-responsive promoter and with an adenovirus expressing a tetracyclin-transactivator. Individual expression of pp65 or pp71 did not affect CD81 cell surface expression (Fig 3D). Similarly, CD81 expression was not affected by overexpression of the major viral glycoprotein B (gB) or the two major IE proteins IE1 and IE2 (Fig 3D). These data eliminate five obvious candidates immediately present in the infected cell either due to abundant presence in the virion (pp65, pp71, gB) or due to IE expression kinetics.

Many viruses downregulate their host cell receptors during entry [51]. Interestingly, CD81 has been identified as a co-receptor for the hepatitis C virus (HCV). It has been shown that the HCV glycoprotein E2 binds to the large extra cellular loop of CD81 [52–55]. To further elucidate the effect of HCMV infection on the cell surface expression of CD81 over time, we analyzed the cell surface expression at 2, 4, 8 and 24 hpi of HFFs infected with AD169 with an MOI of 3. A steady and constant decrease of cell surface CD81 expression is visible shortly after viral entry starting at 2 hpi and continuing throughout the experiment (Fig 3E). These data suggest that CD81 is affected both immediately upon entry of HCMV and later in infection due to two independent mechanisms.

### MβCD blocks HCMV infection and alters TEM proteins

**Fig 4:**
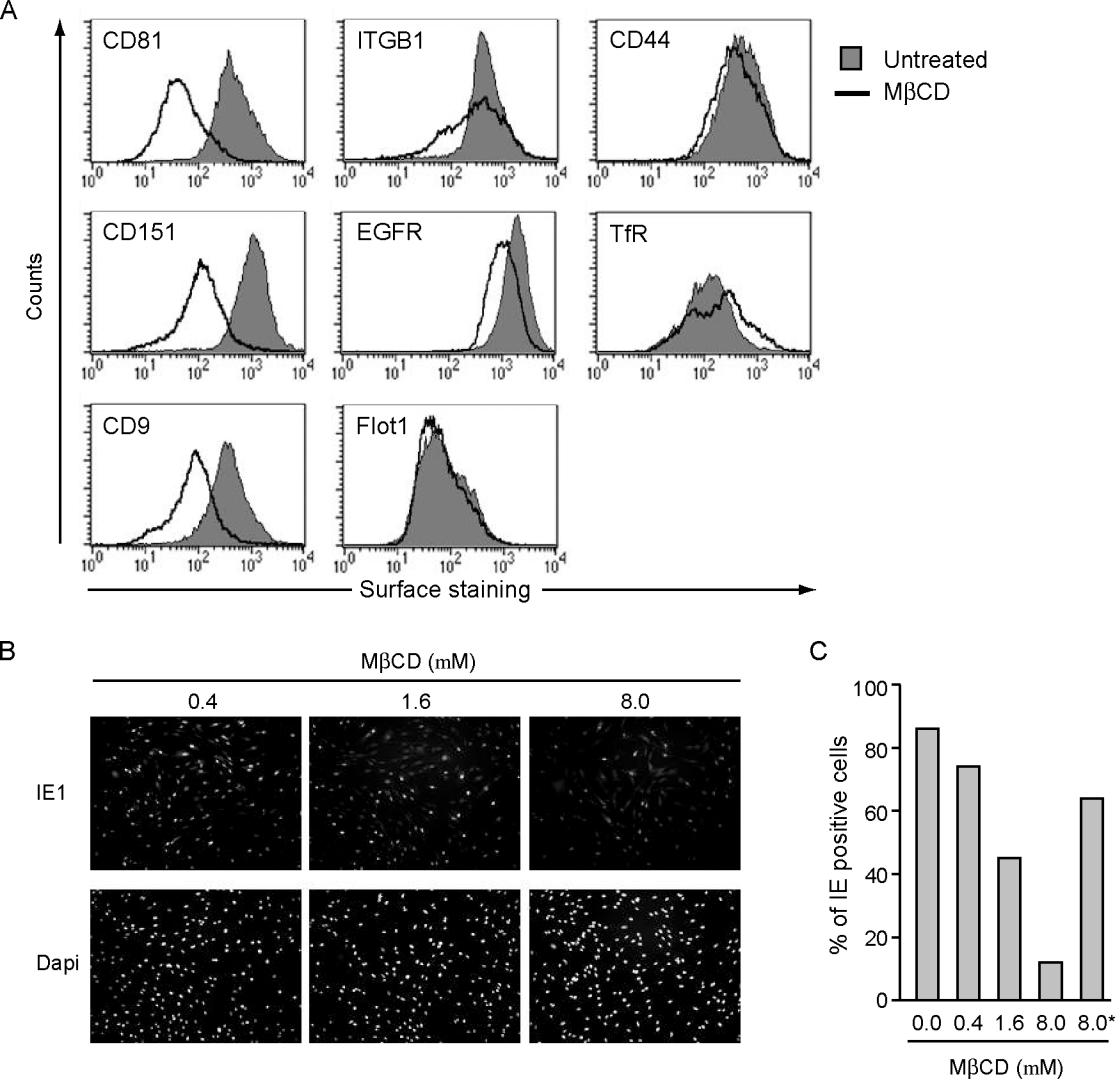
MβCD alters the levels of tetraspanin-enriched membrane microdomains-associated proteins and blocks HCMV infection. (A) HFFs were treated with 8.0 mM MβCD for 1h, then the cells were fixed and cell surface expression of the indicated proteins was determined by flow cytometry. (B) HFFs were incubated with increasing concentrations of MβCD for 1h and subsequently infected with AD169 at an MOI of 3. The cells were fixed at 8 hpi and stained for IE1 and DAPI using IFA. (C) The IE1 positive cells shown in (B) were counted and the percentage of IE1 positive cells was calculated in relation to the DAPI-positive cells. *Indicates cells that were treated with 8 mM MβCD and with cholesterol. Experiments were repeated three times and the result of one representative experiment is shown.

CD81 localizes to TEMs that form a loosely connected network called the tetraspanin-web [56, 57]. TEMs are distinct from lipid rafts, which are more rigid structures that mostly contain GPI-linked proteins and consist almost exclusively of highly hydrophobic lipids, such as cholesterol. This makes lipid rafts triton-X100 insoluble [58], while TEMs are soluble in triton-X100. It was previously demonstrated that HCMV-entry was blocked by treatment with the cholesterol chelator MβCD. This block was reversed when the culture medium was spiked with cholesterol [59]. Given the known importance of cholesterol in the formation of lipid rafts, it was concluded that the effect of MβCD was due to the disruption of lipid rafts. However, it has been shown that tetraspanins interact with cholesterol and this interaction is thought to play an important role in the organization of TEMs [60, 61]. Indeed, MβCD has been shown to disrupt TEMs [61]. Additionally, tetraspanins within TEMs associate with a multitude of transmembrane proteins including integrins which are known to be involved in HCMV-entry [62]. Therefore we hypothesized that TEMs play a role in HCMV-entry. We first studied the effect of MβCD on TEMs in HFFs. We observed a reduction in cell surface levels of the tetraspanins CD81, CD151 and CD9 in uninfected HFFs treated with 8 mM MβCD at 37°C for 1h (Fig 4A). In addition, we detected an MβCD-induced downregulation of the TEM-associated proteins integrin β1 (ITGB1) and EGFR (Fig 4A). EGFR has been implicated in HCMV entry [44], although this data has been disputed [63, 64]. We did not observe an effect of MβCD-treatment on the cell surface expression of lipid raft markers Flotillin1 (Flot1) and CD44 (Fig 4A). However, this staining does not rule out a redistribution of the proteins due to the disruption of lipid raft during MβCD-treatment, so lipid rafts cannot be conclusively eliminated as being involved in HCMV entry. The transferrin receptor (TfR), which is not associated with membrane microdomains, was used as a control and was not affected by MβCD-treatment either (Fig 4A). Nevertheless, we can conclude that MβCD-treatment downregulates TEM-associated proteins from the cell surface and disrupts these domains. To study the effect of TEM disruption on HCMV infection HFFs were infected with AD169 in the presence of increasing concentrations of MβCD. This resulted in a block of HCMV-entry as indicated by a decreasing percentage of cells that showed IE1 nuclear staining in our IFA (Fig 4B). We displayed the number of IE1-positive cells in the presence of an increasing amount of MβCD in a bar-graph (Fig 4C). The MβCD-induced block in HCMV-entry was reversible by the addition of cholesterol to the culture medium (Fig 4C, last bar). All together, these data show that MβCD treatment of HFFs results in the downregulation of TEM-associated proteins and inhibits HCMV entry.

### HCMV downregulates multiple tetraspanins

**Fig 5:**
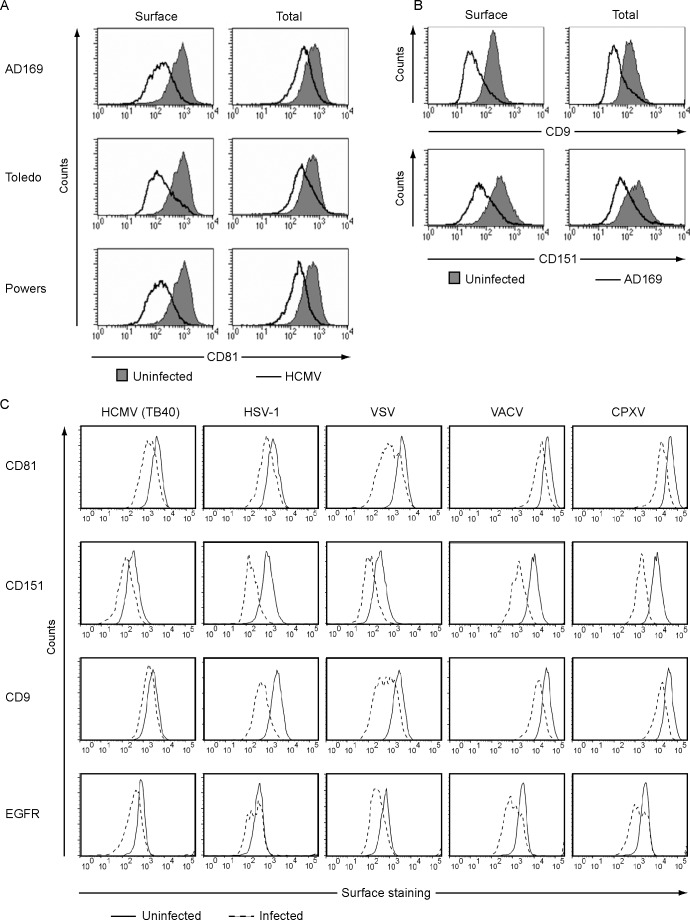
HCMV downregulates tetraspanins. (A) HFFs were infected with the HCMV strains AD169, Toledo, or Powers at an MOI of 3. At 24 hpi the cells were stained for cell surface CD81 expression, or fixed and permeabilized and stained for total CD81 protein expression using flow cytometry. (B) HFFs were infected with AD169 for 24h at an MOI of 3 and the cell surface and total protein expression of CD9 and CD151 was measured as described above. (C) HFFs were left uninfected or infected with HCMV TB40 (MOI of 5), HSV-1 (MOI of 10), VSV (MOI of 10), VACV (MOI of 5) and CPXV (MOI of 5). At 24 hpi the cells were fixed and stained for surface expression of CD81, CD151, CD9 or EGFR using flow cytometry. Before analysis HCMV-, VACV- and CPXV-infected HFFs were gated for GFP. Experiments were repeated two to three times and one representative experiment is shown.

As shown in Fig 2C the laboratory adapted HCMV strain AD169 as well as two tested low-passages isolates, Toledo and Powers, downregulate CD81 from the cell surface. Subsequently, we set to investigate the HCMV-induced reduction of total CD81 levels using the same strains. Cell surface downregulation was confirmed in non-permeabilized cells, however we also observed a clear reduction in total CD81 levels, indicating that the virus induces protein degradation (Fig 5A). Since CD81 forms microdomains with other tetraspanins we investigated whether HCMV-entry affects the cell surface expression of tetraspanins known to form complexes with CD81. To this end, we analyzed the surface levels of the tetraspanins CD9 and CD151 on HFFs infected with HCMV AD169. At 24 hpi, cell surface levels of CD9 and CD151 were reduced in infected cells (Fig 5B). Therefore, we conclude that HCMV downregulates certain tetraspanins from the cell surface. In addition, we observed a reduction in total CD9 and CD151 protein levels (Fig 5B). These data indicate that HCMV infection leads to the internalization and degradation of the tetraspanins CD81, CD9, and CD151.

To determine whether tetraspanins are specifically downregulated by HCMV or generally downregulated by any virus entering HFFs, we determined cell surface levels of the tetraspanins CD81, CD151 and CD9 as well as EGFR 24 hpi with HCMV TB40; HSV-1, a human herpesvirus from the α-herpesvirus subfamily; vesicular stomatitis virus (VSV), an enveloped negative strand RNA virus of the family *rhabdoviridae*; vaccinia virus (VACV), a DNA virus from the orthopox family; and cowpox virus (CXPV), a cloosely related poxvirus. Infection with HCMV TB40 and HSV-1 downmodulated the tetraspanins CD81, CD151, and CD9, as well as EGFR (Fig 5C). This indicates that the downregulation of tetraspanins, and possibly their role in viral entry, is likely to be conserved across herpesviruses. Interestingly, infection of cells with VSV, which is known to be infectious and pathogenic in humans, also led to the downregulation of CD9, CD81, CD151 and EGFR (Fig 5C). The role of tetraspanins in VSV infection is currently unclear due to conflicting reports showing different effects of tetraspanin expression on VSV entry and infection [65, 66]. In addition, the orthopoxviruses VACV and CPXV both induced surface downregulation of the indicated molecules (Fig 5C). EGFR activation has been shown to be essential for VACV-infection of HeLa cells [67]. However, the role of tetraspanins in orthopoxvirus entry has not been studied to date. In conclusion, these data show downregulation of CD9, CD81, CD151, and EGFR upon infection with two herpesviruses, two orthopoxviruses and a negative strand RNA virus, suggesting that the reduction of cell surface expression of these molecules is common upon infection.

### Tetraspanins CD81, CD9 and CD151 are necessary for HCMV-entry

**Fig 6:**
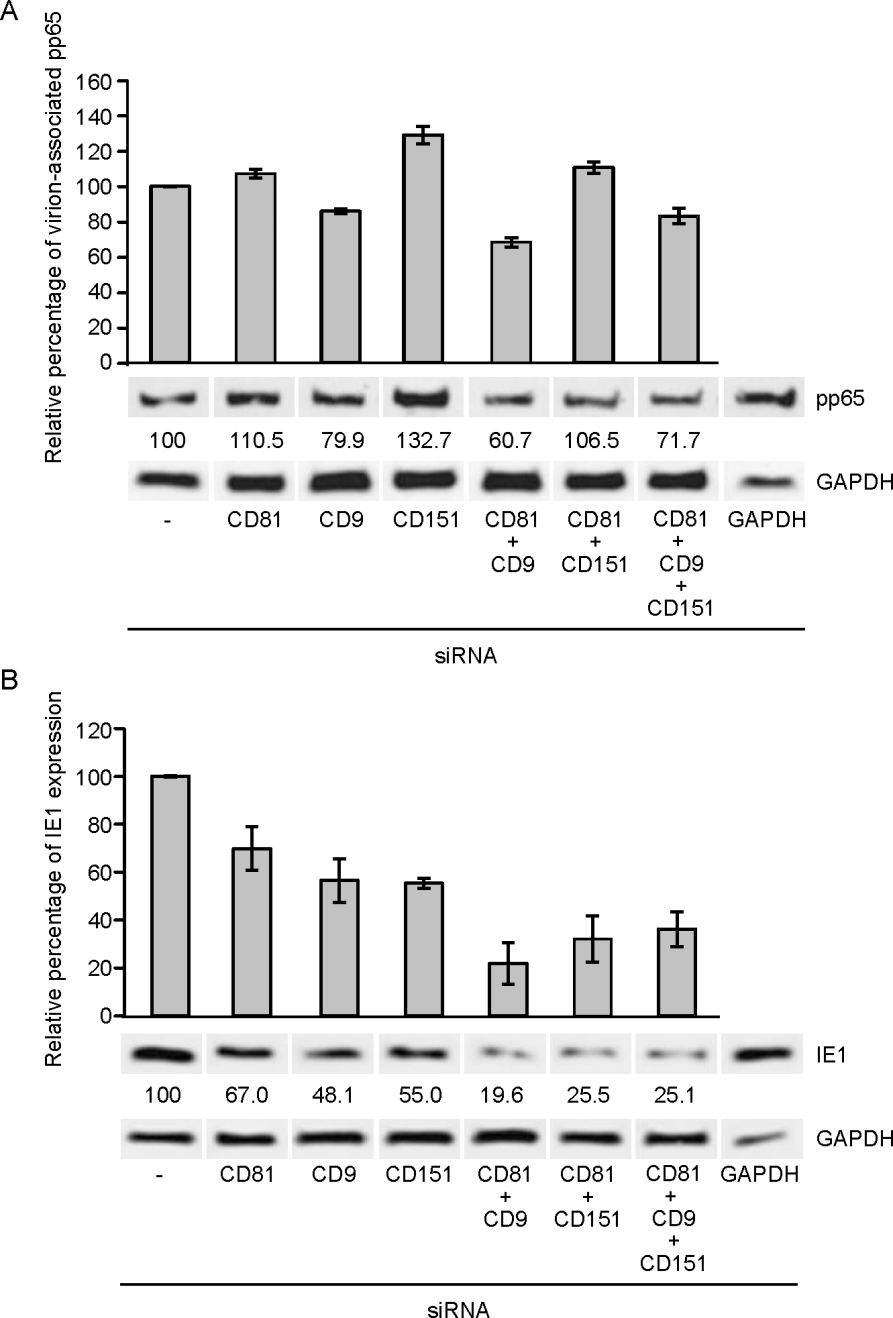
Tetraspanins CD81, CD9 and CD151 play a redundant role in HCMV-entry. HFFs were transfected with the indicated siRNAs for 64h. Control cells were left untransfected. (A) HCMV attachment to these cells was studied directly after incubating the cells for 1h with AD169 at an MOI of 3 on ice. The cells were lysed and samples were analyzed for pp65 levels of attached virions using SDS-PAGE and Western blot. The graph shows relative pp65 levels in every sample compared to untransfected HCMV-infected cells that were set to 100%. (B) Transfected HFFs were incubated with AD169 at an MOI of 3 for 1h at ice and subsequently moved to 37°C for 1h. The cells were washed with citric acid wash buffer to inactivate HCMV virions at the cell surface and infection proceeded for another 8h after which the cells were harvested and lysed. The samples were analyzed for IE1 expression using SDS-PAGE and Western blot. The graph shows relative IE1 expression in every sample compared to untransfected HCMV-infected cells that were set to 100%. Shown are the means ± standard error of the mean of three independent experiments. Experiments were repeated three times, one representative experiment is shown.

The data described above show that HCMV-infection results in the downregulation of multiple tetraspanins (Fig 5). In addition, MβCD also causes downregulation of tetraspanins from the cell surface and the compound blocks HCMV entry (Fig 4). Tetraspanins were shown to function as co-receptors for a range of different viruses [68–74] and studies showed that pretreatment of cells with antibodies specific to single tetraspanins led to the inhibiting of viral entry [72–74]. To further investigate the role of tetraspanins in HCMV entry, we degraded the mRNA of the tetraspanin family members CD81, CD9, and CD151 using specific siRNAs prior to infecting HFFs with HCMV. Cells were transfected with single siRNAs and various combinations to control for redundant roles of the tetraspanins in HCMV entry. The reduction of tetraspanin protein expression was confirmed by flow cytometry (S1 Fig). Compared to non-transfected HFFs, the cells transfected with siRNAs against CD81, CD9, CD151, and combinations thereof displayed noticeably reduced expression levels of the indicated proteins at the cell surface (S1 Fig). Viral attachment was examined by incubating the siRNA-transfected HFFs with AD169 at 4°C for 1h, after which we monitored the presence of the tegument protein pp65 in whole cell lysates using Western blot (Fig 6A). We observed a 39.3% decrease in virion absorption when the protein levels of both CD9 and CD81 were downmodulated. However, we only observed a 28.3% decrease in pp65 levels when all three investigated tetraspanins were targeted and we even saw in increase in HCMV adsorption when we used siRNA specific for CD81 and CD151 (Fig 6A**)**, so we did not observe a consistent phenotype. Next we analyzed immediately early gene expression of AD169 in HFFs after virus infection by staining for IE1 protein expression after 9 hours of incubation at 37°C. We observed a 50% reduction in IE1 expression when single tetraspanins were downmodulated (Fig 6B). Moreover, when combinations of at least two of the tetraspanins (CD81 and CD9; CD81 and CD151; or CD9, CD81 and CD151) were targeted, IE1 expression was reduced by 75% (Fig 6B). This a noticeable reduction of immediate early gene expression is most likely indicative of reduced viral entry into the cells. However, it is possible that other viral entry steps in between viral penetration and into the cell and immediate early gene expression could also be affected by the targeted silencing of selected tetraspanins. Taken together, these results imply that tetraspanins are not involved in the attachment of the virus but are important for productive infection of HFFs with HCMV. The most prominent inhibition the HCMV entry pathway was observed when multiple tetraspanins were downmodulated indicating redundant functions for tetraspanins during virus infection.

## Discussion

By comparing protein abundance in membrane fractions of human fibroblasts infected with HCMV to uninfected cells we identified several transmembrane or membrane-associated proteins that were consistently downregulated during the early stages of HCMV infection. The proteomics approach used here was not intended to provide a comprehensive picture of all proteins that change in HCMV-infected cells, but to identify novel proteins affected by HCMV in a systematic fashion. This approach resulted in new leads for membrane proteins involved in viral entry. We particularly focused on the downregulation of CD81 and other tetraspanins by HCMV and demonstrated that TEMs are involved in the entry of HCMV.

Since HCMV is capable of entering multiple cell types, one can assume that many different or ubiquitous host cell proteins are used to enter the cells. The downregulation of CD81 was found to be bi-phasic and did not require viral gene expression early in infection. At later time points of infection, however, continued downregulation of CD81 required expression of IE and E, but not L genes (Fig 3A and 3B). Viruses often downregulate their receptors after entering into the cells either as a bystander effect of the entry process, to block superinfection of the cells by the same virus, or to facilitate egress by eliminating the possibility of the viral glycoproteins to interact with the cellular receptor [51]. For instance, HSV-1 gD binds to and downregulates the herpesvirus entry mediator (HVEM) during its entry [75]. Therefore, we hypothesized that entry-mediated downregulation of CD81 might be an indication that CD81 is part of complex that contains receptors for HCMV entry.

CD81 is a tetraspanin, like CD151 and CD9 that were also downregulated upon HCMV infection (Fig 5B). Tetraspanins are a small family of cell surface proteins with 33 human members, each one containing four conserved transmembrane domains, characteristic extracellular loops, and short cytoplasmic domains [76]. These proteins are known as molecular organizers of multi-protein membrane complexes that influence cell proliferation, fusion, signaling, and migration [77]. These microdomains are called TEMs and are distinct from classical lipid rafts and caveolae [78]. Treatment of HFFs with MβCD resulted in the downregulation of tetraspanins CD151, CD9, CD81, and of the TEM-associated proteins EGFR and ITGB1, indicating that the TEMs were disrupted. It is likely that this disruption was causative for the observed HCMV entry defect into the cells (Fig 4B), although we cannot exclude potential bystander effects on other cell membrane domains like lipid rafts that might also be disrupted by MβCD treatment and might therefore contribute to the observed phenotype. A recent publication showed that silencing CD151 expression using specific siRNAs lead to reduced entry, but not binding, of HCMV to the cells [79]. Using siRNAs we showed that targeting individual tetraspanins only had a moderate influence on HCMV immediate early gene expression. However, downmodulation of two or more tetraspanin resulted in a substantial and reproducible reduction of HCMV IE1 gene expression most likely as the result of reduced virus entry (Fig 6B). Attachment of virions to the target cells was largely unaffected, however (Fig 6A). Given the redundancy of tetraspanin function in HCMV entry it seems unlikely that tetraspanins act like *bona fide* receptors of HCMV entry. Instead, it seems more plausible that tetraspanins contribute to HCMV entry by condensing the actual HCMV receptors in TEMs.

Published reports imply a role for EGFR, platelet-derived growth factor-α receptor (PDGFR-α), integrin αvβ3, and cellular integrins in HCMV-entry of cell lines [44, 59, 80–82]. Cobbs *et al*. demonstrated that HCMV entry into lung fibroblasts and glioma cells depends on PDGFR-α and results in the phosphorylation of the receptor [80, 83]. CD9 is known to associate with PDGFR-α [84] and tetraspanins residing in TEMs haven been shown to influence signaling of many receptor tyrosine kinases (reviewed in [85]). It is possible that TEM-mediated enhancement of receptor tyrosine kinase signaling is important for HCMV entry. However, a mutant form of PDGFR-α deleted for the cytoplasmic kinase domain was still able to enhance HCMV infection of fibroblasts, indicating the signaling by PDGFR-α is not required for HCMV entry [82]. Given the known association of tetraspanins with the previously proposed HCMV receptors we deem it more likely that tetraspanins facilitate the clustering of those host factors in TEMs, thereby providing a platform for HCMV entry.

Work by Wang *et al*. implicated an involvement of lipid rafts in HCMV entry of lung fibroblasts and that the widely expressed EGFR as well as integrin αVß3 proteins act as co-receptors in this process [44, 59]. Integrins have been shown to regulate the recycling and activation of grow factor receptors [86] and the interaction between these proteins are regulated in lipid rafts [87]. Our data do not exclude an involvement of lipid rafts in HCMV entry. Treatment of fibroblasts with MβCD did not affect the surface levels of lipid raft proteins Flot1 and CD44 (Fig 4A), however, we did not rule out disruption of lipid rafts composition or protein signaling in this experiment. Vice versa, EGFR and integrins are known to associate with TEMs in the cell membrane [56], so the results published by Wang *et al*. do not rule out an involvement of TEMs [59].

The tetraspanins CD63, CD81 and CD151 have been previously implicated in the viral entry of multiple human viruses, including human papilloma virus type 16, 18, and 31 [88–90] and human T cell leukemia virus type 1 (HTLV-1) [71]. The same molecules are known to act as co-receptors for HCV [91, 92]. While CD81 alone is not sufficient for HCV entry, a recently published mouse model expressing known human entry receptors demonstrated that CD81 had to be co-expressed with occludin, scavenger receptor type B class I, and claudin 1 for optimal infection of murine hepatocytes [93]. CD81 has also been suggested to play a role in the entry of members of the *retroviridae* family (human immunodeficiency virus type 1 (HIV-1) and HTLV-1) since CD81 and CD82 associate with CD4 [94, 95]. Additionally, tetraspanins have been shown to facilitate infection of white spot syndrome virus [69], Influenza A virus [70], canine distemper virus [72], feline immunodeficiency virus [74], as well as sporozoites from *Plasmodium yoelii* and *Plasmodium falciparum* [61]. In most of these cases, tetraspanins seem to be part of a larger receptor complex assisting in the facilitation of viral entry. The data presented in Fig 5C showed a downregulation of tetraspanins CD81, CD151 and CD9 upon infection with VSV and the orthopoxviruses VACV and CPXV. This suggests that tetraspanins might play a role in the infection of human fibroblasts by these viruses as well, possibly through the clustering of receptors that are essential for entry like we proposed for HCMV. Interestingly, pretreatment of target cells with MβCD has been shown to block VSV entry [96], supporting this hypothesis.

In addition to the tetraspanins we have found the membrane-associated levels of CD98, CD44, caveolin-1 and δ-catenin to be downmodulated upon HCMV-infection. CD98, a type II transmembrane glycoprotein, is the heavy chain of the heteromeric large neutral amino acid transporter. This protein was significantly downregulated from the cell surface of HCMV-infected cells (Fig 2A) and seemed to be degraded since steady state levels were significantly reduced in whole cell lysates of HCMV-infected cells (Fig 2B). In addition to being a solute transporter subunit, CD98 was shown to associate with integrin B subunits thereby activating integrins and promoting cell adhesion [97]. Kaposi's sarcoma-associated herpesvirus (KSHV) forms a multi-molecular complex of integrins (αVβ5, αVβ3, and α3β1), CD98 and cystine/glutamate transporter (xCT) during infection of human dermal microvascular endothelial cells [98]. This complex is essential for the entry and post entry stages of KSHV infection and the CD98-xCT complex seems to facilitate the fusion of viral envelopes with the cellular membrane [99]. A recent publication demonstrated co-localization of KSHV, CD98, integrins αVβ3, αVβ5, α3β1, and the lipid raft marker cholera toxin B on a fibrosarcamo cell line before entry of the virus [100]. Integrin αVß3 and lipid rafts were demonstrated to play a role in HCMV entry [81]. Together, our finding that CD98 was downmodulated upon HCMV-infection and the above discussed reports make it conceivable that CD98/integrin complexes localizing to lipid rafts are involved in HCMV entry. CD44 is involved in cell-matrix and cell-cell interactions including adhesion, migration and signal transduction and has previously been shown to be upregulated on the surface of human embryonic fibroblasts infected with HCMV strain AD169 [101]. In our hands, although transcriptional levels remain unaffected (Fig 2E), CD44 levels are reduced both at the cell surface and in total cell lysates (Fig 2A, 2B and 2D). CD44 is a heparin sulphate proteoglycan (HSPG) also known as extracellular matrix receptor III, hyaluronan receptor and PGP-1. CD44 is a highly glycosylated type I transmembrane protein that exist in at least 17 known isoforms. Thus, it is possible that these different findings are due to differences in antibodies used that recognize different isoforms. Therefore, CD44-downregulation by HCMV might be isoform-specific. Initial viral attachment often involves non-specific interaction of viral glycoproteins with HSPGs present on the cell surface [102], which can be an explanation for CD44 downregulation on infected cells.

Caveolin-1 and δ-catenin are associated with the cytoplasmic membrane and involved in clathrin-dependent and -independent endocytosis, respectively. Total levels of caveolin-1 were strongly reduced in HFFs productively infected with HCMV strains AD169, Toledo and Powers (Fig 2). Interestingly, HCMV virions have been shown to induce caveolae formation in non-productively infected cytotrophoblasts and caveolin-1 co-localizes with gB during this process [103]. Thus, it is conceivable that expression of gB in HCMV-infected fibroblasts was responsible for the observed reduction of caveolin-1 levels. The abundance of the membrane-associated protein δ-catenin was also reduced in HCMV-infected cells (Fig 1). However, due to the lack of suitable antibodies or low levels of expression, we did not study δ-catenin in great detail. δ-catenin is involved in clathrin-mediated endocytosis, a process that is essential for the entry of multiple viruses [67]. Catenin-associated cadherins were previously shown to co-localize with HCMV glycoprotein gpUS9 [104], so it is possible that catenin-downregulation results from the activity of early glycoproteins such as US9 on cadherins.

In conclusion, TEMs have been linked to the entry of numerous viruses spanning multiple different RNA- and DNA-virus family members. However, to our knowledge this is the first report showing that the tetraspanins CD81 and CD9 play a crucial role in the entry of a herpesvirus, whereas CD151 has been implicated in HCMV entry before [79]. We show that tetraspanins perform redundant functions, which is possibly a reason why it has been difficult to unequivocally demonstrate their involvement in herpesviral entry. While our data do not rule out the involvement of lipid rafts or caveolae in the entry of HCMV, they strongly suggest that TEMs need to be considered when studying the role of membrane microdomains in herpesviral entry.

## Supporting information

S1 Fig Tetraspanin levels in HFFs transfected with specific siRNAs. HFFs cells were transfected with the indicated combinations of siRNAs for 64h. After that the cells were fixed and stained for cell surface expression of CD81 (A), CD9 (B) and CD151 (C) expression using flow cytometry.
(TIF)

S1 Table 2D-LC-MS/MS results experiment 1. Mock and AD169-infected HFFs were analyzed using tandem 2D-LC-MS/MS at 24h post infection. The algorithm ASAPRatio was used to analyze chances in protein abundance.
(XLSX)

S2 Table 2D-LC-MS/MS results experiment 2. Mock and AD169-infected HFFs were analyzed using tandem 2D-LC-MS/MS at 24h post infection. The algorithm ASAPRatio was used to analyze chances in protein abundance.
(XLSX)

S3 Table 2D-LC-MS/MS results experiment 3. Mock and AD169-infected HFFs were analyzed using tandem 2D-LC-MS/MS at 24h post infection. The algorithm ASAPRatio was used to analyze chances in protein abundance.
(XLSX)

S4 Table 2D-LC-MS/MS results of experiment 1–3 combined. This table combines the results, changes in abundance of the described membrane proteins, of the 3 experiments that were performed on AD169-infected cells.
(XLSX)
