# Supplementary figures and images for: Quantitative membrane proteomics reveals a role for tetraspanin enriched microdomains during entry of human cytomegalovirus
Source: PLoS One. 2017 Nov 9;12(11):e0187899. doi: 10.1371/journal.pone.0187899 (PMC5679760; doi:10.1371/journal.pone.0187899)

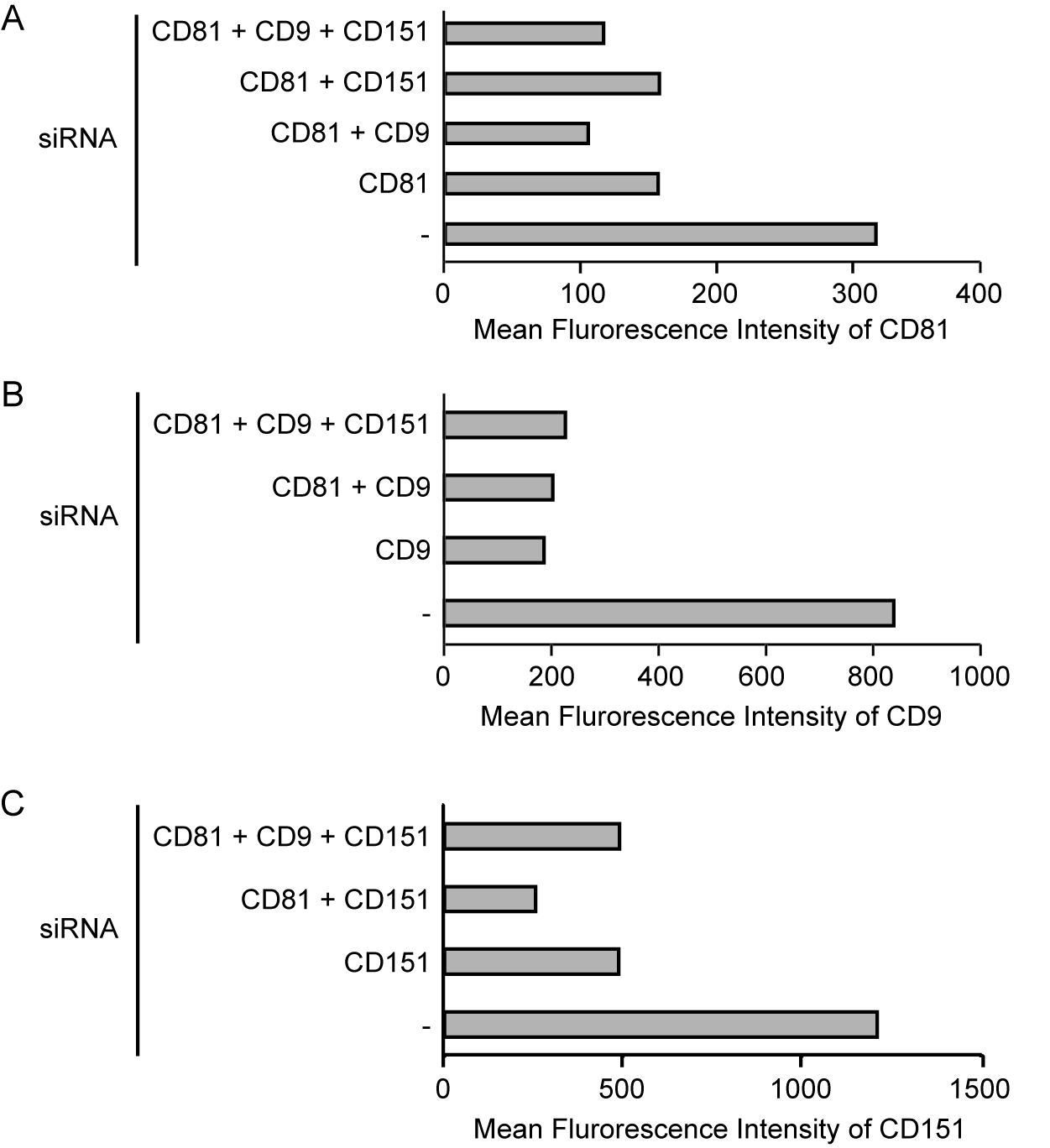

Supplement: S1 Fig — HFFs cells were transfected with the indicated combinations of siRNAs for 64h. After that the cells were fixed and stained for cell surface expression of CD81 (A), CD9 (B) and CD151 (C) expression using flow cytometry. (TIF) [file pone.0187899.s001.tif]
